# Risk of Psychiatric Disorders Among Spouses of Patients With Cancer in Denmark and Sweden

**DOI:** 10.1001/jamanetworkopen.2022.49560

**Published:** 2023-01-05

**Authors:** Kejia Hu, Qianwei Liu, Krisztina D. László, Dang Wei, Fen Yang, Katja Fall, Hans-Olov Adami, Weimin Ye, Unnur A. Valdimarsdóttir, Jiong Li, Fang Fang

**Affiliations:** 1 Unit of Integrative Epidemiology, Institute of Environmental Medicine, Karolinska Institutet, Stockholm, Sweden; 2 Department of Global Public Health, Karolinska Institutet, Stockholm, Sweden; 3 Clinical Epidemiology and Biostatistics, School of Medical Sciences, Örebro University, Örebro, Sweden; 4 Department of Medical Epidemiology and Biostatistics, Karolinska Institutet, Stockholm, Sweden; 5 Clinical Effectiveness Group, Institute of Health and Society, University of Oslo, Oslo, Norway; 6 Department of Epidemiology, Harvard T.H. Chan School of Public Health, Boston, Massachusetts; 7 Center of Public Health Sciences, Faculty of Medicine, University of Iceland, Reykjavík, Iceland; 8 Department of Clinical Medicine & Department of Clinical Epidemiology, Aarhus University, Aarhus, Denmark

## Abstract

**Importance:**

There is emerging evidence that spouses of patients with cancer may have a higher prevalence of mental illness, but these studies have been limited by pre-post designs, focus on a single mental illness, and short follow-up periods.

**Objectives:**

To assess the overall burden of psychiatric disorders among spouses of patients with cancer vs spouses of individuals without cancer and to describe possible changes in this burden over time.

**Design, Setting, and Participants:**

This population based cohort study included spouses of patients with cancer (diagnosed 1986-2016 in Denmark and 1973-2014 in Sweden; exposed group) and spouses of individuals without cancer (unexposed group). Members of the unexposed group were individually matched to individuals in the exposed group on the year of birth, sex, and country. Spouses with and without preexisting psychiatric morbidity were analyzed separately. Data analysis was performed between May 2021 and January 2022.

**Exposures:**

Being spouse to a patient with cancer.

**Main Outcomes and Measures:**

The main outcome was a clinical diagnosis of psychiatric disorders through hospital-based inpatient or outpatient care. Flexible parametric models and Cox models were fitted to estimate hazard ratios (HRs) with 95% CIs, adjusted for sex, age and year at cohort entry, country, household income, and cancer history.

**Results:**

Among 546 321 spouses in the exposed group and 2 731 574 in the unexposed group who had no preexisting psychiatry morbidity, 46.0% were male participants, with a median (IQR) age at cohort entry of 60 (51-68) years. During follow-up (median, 8.4 vs 7.6 years), the incidence rate of first-onset psychiatric disorders was 6.8 and 5.9 per 1000 person-years for the exposed and unexposed groups, respectively (37 830 spouses of patients with cancer [6.9%]; 153 607 of spouses of individuals without cancer [5.6%]). Risk of first-onset psychiatric disorders increased by 30% (adjusted HR, 1.30; 95% CI, 1.25-1.34) during the first year after cancer diagnosis, especially for depression (adjusted HR, 1.38; 95% CI, 1.30-1.47) and stress-related disorders (adjusted HR, 2.04; 95% CI, 1.88-2.22). Risk of first-onset psychiatric disorders increased by 14% (adjusted HR, 1.14; 95% CI, 1.13-1.16) during the entire follow-up, which was similar for substance abuse, depression, and stress-related disorders. The risk increase was more prominent among spouses of patients diagnosed with a cancer with poor prognosis (eg, pancreatic cancer: adjusted HR, 1.41; 95% CI, 1.32-1.51) or at an advanced stage (adjusted HR, 1.31; 95% CI, 1.26-1.36) and when the patient died during follow-up (adjusted HR, 1.29; 95% CI, 1.27-1.31). Among spouses with preexisting psychiatric morbidity, the risk of psychiatric disorders (first-onset or recurrent) increased by 23% during the entire follow-up (adjusted HR, 1.23; 95% CI, 1.20-1.25).

**Conclusions and Relevance:**

In this cohort study of 2 populations in Denmark and Sweden, spouses of patients with cancer experienced increased risk of several psychiatric disorders that required hospital-based specialist care. Our results support the need for clinical awareness to prevent potential mental illness among the spouses of patients with cancer.

## Key Points

**Question:**

Do spouses of patients with cancer have an increased risk of psychiatric disorders compared with spouses of individuals without cancer?

**Findings:**

In this cohort study of more than 3 million individuals, 6.9% of spouses of patients with cancer developed a psychiatric disorder during follow-up, compared with 5.6% of spouses of individuals without cancer. The risk was statistically significantly increased among spouses of patients with cancer vs spouses of individuals without cancer.

**Meaning:**

In this study, spouses of patients with cancer had increased risk of psychiatric disorders, suggesting that they should be included in the surveillance and counseling of patients with cancer.

## Teaser

This cohort study assesses the overall burden of psychiatric disorders among spouses of patients with cancer.

## Introduction

A diagnosis of cancer is severely stressful for the patients and their families. Particularly, the emotional, physical, and financial burden during the diagnosis, treatment, disease progression, and, often, the death of the patient with cancer may affect the mental health of the spouses.^1,2,3,4^ Some studies have suggested that spouses of patients with cancer might experience more psychiatric symptoms, including depression and anxiety, even when compared with the patients themselves.^5,6,7^ As the primary source of social support and caregiving, spouses are important in the prognosis and quality of life of patients with cancer. Studies have for instance shown that married patients with cancer have better survival than nonmarried patients.^8^

Most existing studies have used self-reported questionnaire data and cross-sectional design to document the burden of mental illness among spouses of patients with cancer.^6,7,9,10,11^ To our knowledge, there are currently 2 population-based studies on this topic.^12,13^ One study^12^ used Swedish health register data and showed an increased risk of psychiatric disorders after cancer diagnosis, compared with before cancer diagnosis, among spouses of patients with cancer. Another study in Japan^13^ suggested a higher risk of depression among spouses of patients with cancer during the first year after cancer diagnosis compared with spouses of individuals without cancer. Population-based studies are, however, rare concerning the entire spectrum of psychiatric disorders, and longer-term follow-ups are needed. Therefore, based on the national population and health registers in Denmark and Sweden, we aimed to test the hypotheses that (1) spouses of patients with cancer have an increased risk of psychiatric disorders, such as substance abuse, depression, anxiety, and stress-related disorders, compared with spouses of individuals without cancer, and (2) the excess risk is more pronounced immediately after cancer diagnosis.

## Methods

### Study Design

We designed a matched cohort study within the Danish-Swedish joint project of stressful life events,^14^ which includes the general Danish population born from 1878 to 2016 and a general Swedish population born from 1973 to 2014 as well as their parents. Among these, we first identified all patients with a first diagnosis of primary malignant neoplasms from 1943 to 2016 according to the Danish Cancer Register (803 085 individuals)^15^ and from 1958 to 2014 according to the Swedish Cancer Register (291 936 individuals).^16^ Through the Danish Civil Registration System (CRS; with information on spousal link since 1986),^17^ a Danish spouse was defined as a registered heterosexual spouse of a patient with cancer, who was 18 years or older at the time of cancer diagnosis. Because we had no spousal link in the Swedish data, we defined a Swedish spouse as someone (1) 18 years or older at the diagnosis of the patient with cancer who (2) shared a biological child with the patient and (3) was not divorced or widowed between birth of the shared child and diagnosis of cancer, through linkage with Swedish Total Population Register (TPR, with information on marital status since 1973)^18^ and Medical Birth Register (MBR, with information on all mothers and 83% fathers of all births since 1973),^19^ complemented by the Swedish Multi-Generation Register.^20^ Such a method of identifying spouses in Sweden has been used in a previous study.^21^ As a result, we defined the follow-up period as 1986 to 2016 in Denmark and 1973 to 2014 in Sweden. The registers used in the present study are public registers in Denmark and Sweden, without specific inclusion or exclusion criteria.^22,23^ This study was approved by the Danish Data Protection Agency, Copenhagen, and the Ethics Review Board in Stockholm. Informed consent is not required for research using register-based health care data according to the General Data Protection Regulation of the European Union. This study followed the Strengthening the Reporting of Observational Studies in Epidemiology (STROBE) reporting guideline.

As we are primarily interested in new-onset psychiatric disorders, we excluded individuals who had a diagnosis of psychiatric disorders or had been exposed (ie, had a spouse with cancer) before the start of follow-up, leaving 546 321 spouses in the final exposed group (eFigure in Supplement 1). The date of diagnosis of the patient with cancer was defined as cohort entry for the exposed group. Using the same definition of spouse, we randomly selected, through incidence density sampling,^24^ 5 individuals who were spouses of individuals without cancer and had no preexisting psychiatric disorders at cohort entry of the exposed spouse, individually matched with each spouse in the exposed group on year of birth, sex, and country (eFigure in Supplement 1). The spouses in the unexposed group had the same cohort entry as their matched exposed spouse. Both groups were then followed up from cohort entry until the first diagnosis of psychiatric disorders, emigration, death, or end of follow-up (December 31, 2016, in Denmark and December 31, 2014, in Sweden), whichever came first. The spouses in the unexposed group were additionally censored if they later became exposed and were treated as members of the exposed group thereafter.

### Measures

#### Cancer Diagnosis

The cancer registers in Denmark and Sweden are used as an important tool to monitor cancer incidence and survival and contain almost all incident cases of malignant neoplasms since their establishment.^15,16^ The registers are also considered to be of good quality; for instance, approximately 99% of all the cancer cases are morphologically verified in the Swedish Cancer Regsiter.^16^ In addition to any cancer, we separately analyzed the 20 most common cancer sites and types according to a previous study (eTable 1 in Supplement 1).^21^ We also classified cancer stage as localized (any T/N0/M0), regional spread or advanced (any T/N+/M0 or any T/any N/M+), or unknown stage according to the European Network of Cancer Registries Condensed TNM Scheme.^25^

#### Psychiatric Disorders

We identified any first clinical diagnosis of psychiatric disorders that required inpatient or outpatient secondary care during the follow-up of the spouses in the exposed and unexposed groups from the Danish National Patient Registry (since 1977),^26^ the Danish Register of Causes of Death (since 1875),^27^ the Danish Psychiatric Central Research Register (since 1970),^28^ the Swedish National Patient Register (since 1973),^29^ and the Swedish Cause of Death Register (since 1952),^30^ in which diagnoses made in relation to a hospital visit or causes of death are recorded using *International Classification of Diseases *(*ICD*) codes. We used *ICD* eighth edition (*ICD-8*) codes 291 and 295 to 315; *ICD* ninth edition (*ICD-9*) codes 291, 292, and 295 to 319; and *ICD* tenth edition (*ICD-10*) codes F10 to F99 to identify psychiatric disorders. To alleviate the concern of potential misdiagnosis, we focused on the primary diagnosis of each hospital visit in this definition. In addition to any psychiatric disorder, we also separately analyzed a few common psychiatric disorders, such as substance abuse, depression, anxiety, and stress-related disorders (eTable 2 in Supplement 1).

#### Covariates

We collected data on year of birth and sex from the Danish CRS and the Swedish TPR and data on household income and educational attainment from the Danish Integrated Database for Longitudinal Labor Market Research (since 1980),^31^ the Swedish Register of Incomes and Taxes (since 1972), and the Swedish Education Register (since 1990). Missing data for education and income at the cohort entry were replaced by the information of the closest year within 5 years before cohort entry and deemed unknown if no information was found. We further collected data on the history of cancer for spouses in both the exposed and unexposed groups from the cancer registers. To reflect lifestyle factors, smoking status and body mass index (BMI [calculated as weight in kilograms divided by height in meters squared]) during the first pregnancy were retrieved from the Danish and Swedish MBRs (only available for female participants). A full list of hospital diagnoses was available for the Danish participants to calculate Charlson Comorbidity Index (CCI) as a proxy for general physical health status.^32,33^

### Statistical Analysis

We first fitted flexible parametric models to visualize the time-dependent hazard ratio (HR) of psychiatric disorders in relation to being the spouse of a patient with cancer. We separately estimated HRs during the entire follow-up period as well as during the first year of follow-up, as a high-risk period, using Cox models. No major violation of the proportional hazard assumptions was found for the main variables (eMethods in Supplement 1). All models were adjusted for age and calendar year at cohort entry, sex, country, household income, and history of cancer. Age at cohort entry was fitted using natural cubic splines to adjust for nonlinear confounding.

We performed subgroup analyses by sex, age and calendar year of cohort entry, country, household income, and history of cancer. We added an interaction term between exposure (ie, being spouse to a patient with cancer) and each of the covariates to the multivariate models and used the Wald test to test the statistical significance of the interaction term. We further separately calculated HR of inpatient or outpatient diagnosis of any psychiatric disorder. To assess the different associations of cancer characteristics, we performed subgroup analyses by age and stage at cancer diagnosis as well as cancer sites and types. To address potential residual confounding, we further adjusted for educational attainment, smoking status, BMI, or CCI within subsets of the cohort with available data. Finally, we used information on marital status (time-varying) and presence of children in the household (based on the Danish data) to assess the role of family status in the studied association.

The main analyses were restricted to individuals without preexisting psychiatric morbidity, as we were primarily interested in new-onset psychiatric disorders. To assess the outcomes of cancer diagnosis among spouses with preexisting psychiatric morbidity, we performed a secondary analysis by constructing a separate cohort including spouses in both the exposed and unexposed groups who had a psychiatric disorder before cohort entry, using the same method as in the main analysis. In this cohort, we followed up the exposed and unexposed groups for a hospital visit for any psychiatric disorder, newly diagnosed or previously known, after cohort entry. Furthermore, we assessed the risk of first-onset substance abuse, depression, anxiety, and stress-related disorders in this high-risk group by studying spouses who had previous psychiatric disorders other than each of these disorders. Cox models with the same multivariate adjustment as in the main analysis were fitted.

We used SAS version 9.4 (SAS Institute), Stata version 15.1 (StataCorp), and R version 4.1.1 (package forestplot, version 2.0.1 [R Project for Statistical Computing]) for data management, analysis, and visualization. Complete case analysis was used to deal with the missingness of covariates in all models. Statistical significance was determined based on a 2-sided *P* < .05. Data were analyzed from May 2021 to January 2022.

## Results

We included 546 321 spouses of patients with cancer (exposed group) and 2 731 574 spouses of individuals without cancer (unexposed group) in the main analysis (Table 1). There were slightly more female than male participants (54.0% vs 46.0%), and the median (IQR) age at cohort entry was 60 (51-68) years. Most patients with cancer (69.0%) were diagnosed after 2000 and because of the study design, more participants (70.4%) were Danish residents. Spouses in the exposed group had a slightly higher level of household income and were also more likely to have a cancer history themselves compared with those in the unexposed group. Danish spouses were more likely to be female, older, and to have lower income and education than Swedish spouses (eTable 3 in Supplement 1).

**Table 1.** Baseline Characteristics of the Study Participants

Characteristic	Participants, No. (%)	
	Exposed group: spouses of patients with cancer (n = 546 321)	Unexposed group: spouses of individuals without cancer (n = 2 731 574)^a^
Sex		
Male	251 462 (46.0)	1 257 287 (46.0)
Female	294 859 (54.0)	1 474 287 (54.0)
Age at cohort entry, y		
Median (IQR)	60 (51-68)	60 (51-68)
18-39	40 536 (7.4)	202 815 (7.4)
40-59	219 315 (40.1)	1 096 933 (40.2)
60-79	265 352 (48.6)	1 326 215 (48.6)
80-104	21 118 (3.9)	105 611 (3.9)
Calendar year of cohort entry		
1973-1989	43 048 (7.9)	215 237 (6.4)
1990-1999	126 383 (23.1)	631 914 (23.1)
2000-2009	213 427 (39.1)	1 067 113 (39.1)
2010-2016	163 463 (29.9)	817 310 (29.9)
Country of residence		
Denmark	384 553 (70.4)	1 922 757 (70.4)
Sweden	161 768 (29.6)	808 817 (29.6)
Household income^b^		
Below the lowest tertile	219 660 (40.2)	1 177 648 (43.1)
Between the lowest and middle tertile	110 204 (20.2)	528 201 (19.3)
Above the highest tertile	214 779 (39.3)	1 014 446 (37.1)
Unknown	1678 (0.3)	11 279 (0.4)
History of cancer		
Yes	47 664 (8.7)	208 333 (7.6)
No	498 657 (91.3)	2 523 241 (92.4)
Years of education		
0-9	172 481 (31.6)	877 236 (32.1)
10-14	244 312 (44.7)	1 203 844 (44.1)
≥15	89 775 (16.4)	440 254 (16.1)
Unknown	39 753 (7.3)	210 240 (7.7)

^a^ Individually matched by sex, year of birth, and country of residence. Date of cohort entry was the date of cancer diagnosis for the exposed spouses.

^b^ Household income was classified into 3 groups according to the tertile distribution of each 10-year intervals in each country.

**Figure 1.**
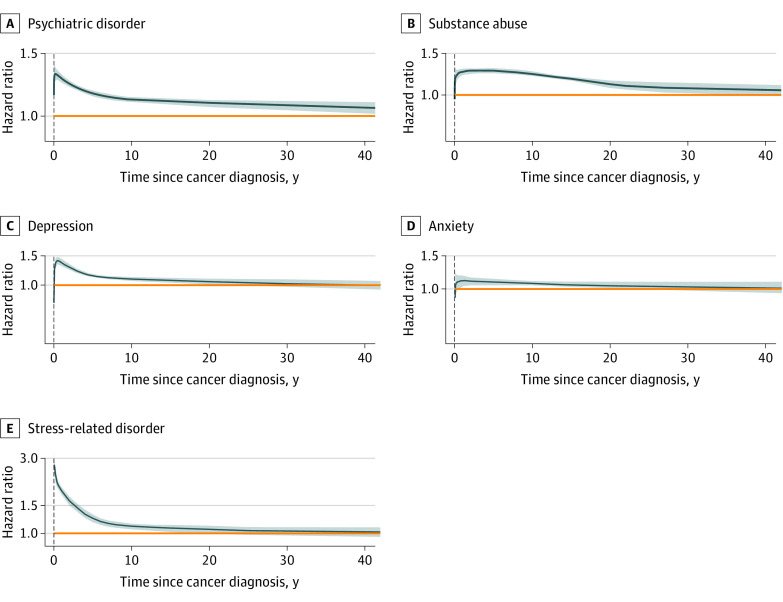
Time-Dependent Hazard Ratios of Any First-Onset Psychiatric Disorder, Substance Abuse, Depression, Anxiety, and Stress-Related Disorder Among Spouses of Patients With Cancer The dark blue lines and shaded areas represent the hazard ratios and 95% CIs. The orange horizontal lines indicate a constant hazard ratio at 1 in each panel. Hazard ratios and associated 95% CIs were estimated from flexible parametric models (Royston-Parmar models), allowing the association of a cancer diagnosis of a spouse with the risk of any psychiatric disorder to vary over time. A spline with 5 degrees of freedom (4 intermediate knots and 2 knots at each boundary, placed at quintiles of distribution of events) was used for the baseline rate, whereas a spline with 3 degrees of freedom was used for the time-varying association. The model was adjusted for sex, age at cohort entry, calendar year at cohort entry, country of residence, household income, and history of cancer.

**Figure 2.**
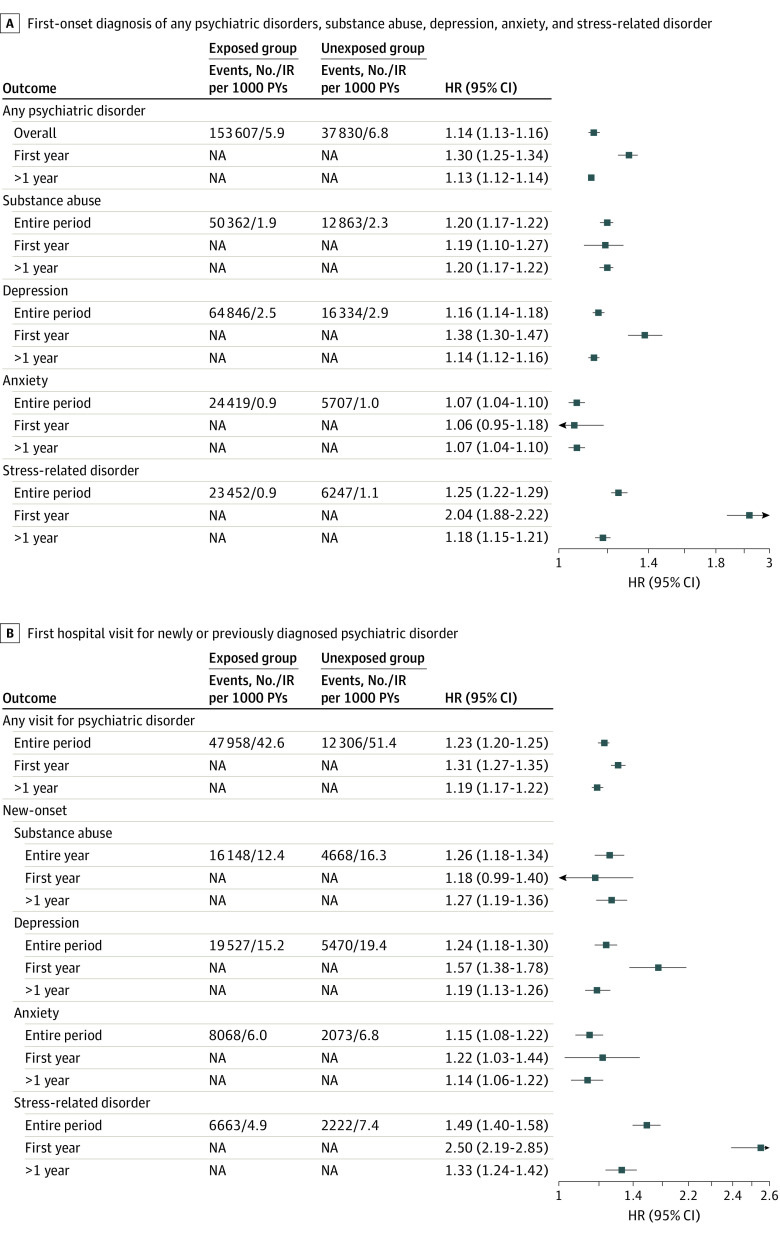
Risk of Psychiatric Disorders for Spouses of Patients With Cancer Among Spouses With or Without Preexisting Psychiatric Comorbidity vs Spouses of Individuals Without Cancer Hazard ratios (HRs) were estimated from Cox models, adjusting for sex, age at cohort entry, calendar year at cohort entry, country of residence, household income, and history of cancer. A, Estimates of first-onset diagnosis of any psychiatric disorder, substance abuse, depression, anxiety, and stress-related disorder among spouses of patients with cancer (exposed group) and spouses of individuals without cancer (matched unexposed group) who had no preexisting psychiatric morbidity. B, All spouses in the exposed and unexposed groups who had previous psychiatric disorders before cohort entry were included and followed up for first hospital visit for any psychiatric disorders, newly or previously diagnosed. Only spouses with a previous psychiatric disorder other than substance use, depression, anxiety, or stress-related disorders were then included and followed up for first-onset of substance abuse, depression, anxiety, or stress-related disorders separately. IR indicates incidence rate; NA, not applicable.

With a median follow-up of 8.4 years for the exposed group and 7.6 years for the unexposed group, we identified a total of 37 830 cases of first-onset psychiatric disorders among the exposed group (6.9%; incidence rate: 6.8 per 1000 person-years) and 153 607 cases among the unexposed group (5.6%; incidence rate: 5.9 per 1000 person-years) spouses. Visualization of time-dependent HRs suggested a constantly increased risk of first-onset psychiatric disorders among spouses in the exposed group during the entire follow-up period, although the magnitude of risk increase decreased over time (Figure 1A). The overall result pattern was similar for the individually studied psychiatric disorders (Figure 1B, C, D, and E). The overall risk of first-onset psychiatric disorders was higher among spouses in the exposed vs unexposed groups (adjusted HR, 1.14; 95% CI, 1.13-1.16) during the entire follow-up (Figure 2A). The excess risk was largely similar for substance abuse, depression, and stress-related disorders, but smaller for anxiety. The HR was highest in the first year after cancer diagnosis for any psychiatric disorder (adjusted HR, 1.30; 95% CI, 1.25-1.34), especially for depression (adjusted HR, 1.38; 95% CI, 1.30-1.47) and stress-related disorders (adjusted HR, 2.04; 95% CI, 1.88-2.22) (Figure 2A).

**Figure 3.**
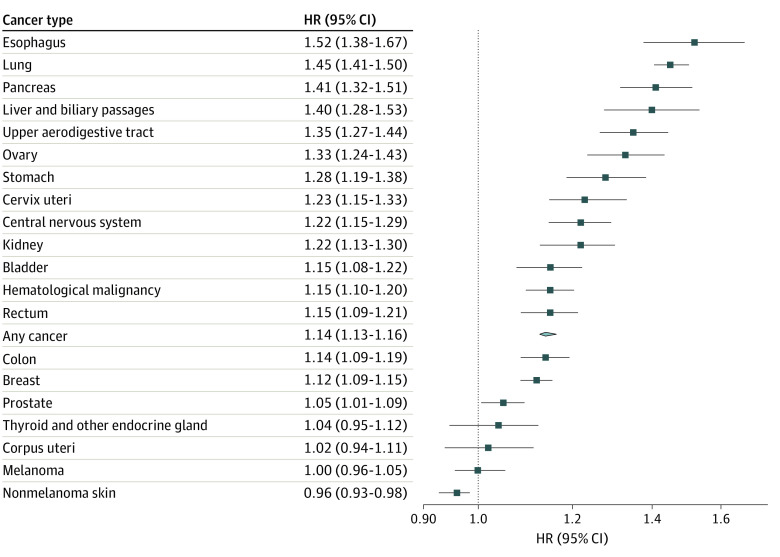
Risk of Any First-Onset Psychiatric Disorder Among Spouses of Patients With Cancer, Stratified by Cancer Site Hazard ratios (HRs) and 95% CIs were estimated from Cox models, adjusting for sex, age at cohort entry, calendar year at cohort entry, country of residence, household income, and history of cancer.

Men and individuals aged 40 to 59 years or 60 to 79 years when exposed to a spousal cancer diagnosis had a slightly greater risk increase of first-onset psychiatric disorders compared with their counterparts (ie, women and individuals in other age groups) (Table 2). Spouses in the exposed group with a low household income had a greater risk increase compared with those with higher household incomes. The HRs were otherwise comparable regardless of the calendar year, country of residence, or history of cancer. Similar to the overall result, the HRs of stratified analyses were greater during the first year of follow-up compared with thereafter. A positive association was noted for most of the studied cancer sites and types (Figure 3). The increased risk of first-onset psychiatric disorders was most prominent for cancer in the esophagus, lung, pancreas, liver, or biliary passages (eg, pancreatic cancer: adjusted HR, 1.41; 95% CI, 1.32-1.51). Finally, the adjusted HR was 1.17 (95% CI, 1.15-1.19) for inpatient diagnosis of psychiatric disorders and 1.13 (95% CI, 1.12-1.15) for outpatient diagnosis.

**Table 2.** Risk of Any First-Onset Psychiatric Disorder Among Spouses of Patients With Cancer, Stratified by Characteristics of Spouses

Characteristic	Spouses of individuals without cancer, entire follow-up		Spouses of patients with cancer					
			Entire follow-up				HR (95% CI)^a^	
	Cases, No.	IR per 1000 PYs	Cases, No.	IR per 1000 PYs	HR (95% CI)^a^	*P* value for interaction^b^	First year of follow-up	>1 Year of follow-up
Sex								
Male	69 693	5.7	17 123	6.7	1.17 (1.15-1.19)	<.001	1.26 (1.19-1.33)	1.16 (1.14-1.19)
Female	83 914	6.2	20 707	6.9	1.12 (1.10-1.14)		1.33 (1.26-1.40)	1.10 (1.08-1.12)
Age at cohort entry, y								
18-39	17 864	5.5	3910	5.9	1.07 (1.04-1.11)	<.001	1.30 (1.13-1.51)	1.06 (1.02-1.10)
40-59	62 942	5.1	15 521	5.9	1.17 (1.15-1.19)		1.23 (1.16-1.31)	1.16 (1.14-1.18)
60-79	67 132	6.8	17 098	7.9	1.15 (1.13-1.17)		1.36 (1.29-1.43)	1.13 (1.11-1.15)
80-104	5669	11.9	1301	12.9	1.07 (1.00-1.13)		1.22 (1.06-1.40)	1.03 (0.97-1.11)
Calendar year at cohort entry								
1973-1989	20 066	4.6	4836	5.3	1.09 (1.06-1.13)	.71	1.54 (1.26-1.87)	1.08 (1.05-1.12)
1990-1999	53 399	5.6	13 275	6.5	1.13 (1.11-1.15)		1.56 (1.42-1.72)	1.12 (1.10-1.14)
2000-2009	62 929	6.7	15 643	7.7	1.16 (1.14-1.18)		1.29 (1.22-1.36)	1.14 (1.12-1.16)
2010-2016	17 213	6.7	4076	7.6	1.14 (1.10-1.18)		1.20 (1.12-1.27)	1.11 (1.07-1.16)
Country of residence								
Denmark	111 712	6.1	28 035	7.0	1.15 (1.13-1.16)	.02	1.33 (1.27-1.39)	1.13 (1.11-1.14)
Sweden	41 895	5.6	9795	6.3	1.12 (1.10-1.14)		1.20 (1.12-1.28)	1.11 (1.08-1.13)
Household income								
Below the lowest tertile	74 511	7.2	17 412	8.8	1.17 (1.15-1.19)	<.001	1.34 (1.28-1.42)	1.15 (1.13-1.17)
Between the lowest and middle tertile	29 611	6.1	7705	6.9	1.14 (1.11-1.17)		1.27 (1.17-1.38)	1.13 (1.10-1.16)
Above the highest tertile	48 567	4.7	12 522	5.2	1.10 (1.07-1.12)		1.22 (1.13-1.31)	1.19 (1.06-1.11)
History of cancer								
Yes	9876	7.9	3037	9.5	1.19 (1.15-1.24)	.09	1.35 (1.22-1.50)	1.16 (1.11-1.22)
No	143 731	5.8	34 793	6.6	1.14 (1.13-1.15)		1.29 (1.24-1.34)	1.13 (1.11-1.14)

Abbreviations: HR, hazard ratio; IR, incidence rate; PY, person-year.

^a^ Estimated from Cox models, adjusting for sex, age at cohort entry, calendar year at cohort entry, country of residence, household income, and history of cancer.

^b^ *P* for interaction was obtained using Wald test to test the statistical significance of interaction term between exposure (ie, being spouse to a cancer patient) and each of the covariates in the specific model.

In the subgroup analysis by other cancer characteristics, a greater risk was found for cancers diagnosed with regional spread or at an advanced stage (adjusted HR, 1.31; 95% CI, 1.26-1.36) compared with cancers diagnosed at a lower stage (eTable 4 in Supplement 1). Further adjustment for educational attainment, comorbidities, and smoking or BMI in early pregnancy did not change the results substantially (eTable 5 in Supplement 1). A greater risk increase was noted for spouses in the exposed group after the death of the patient with cancer (adjusted HR, 1.29; 95% CI, 1.27-1.31) compared with those whose spouse survived the observation period, but the presence of a common child did not change the association (eTable 6 in Supplement 1). Further analyses by sex and marital status during follow-up showed that the increased risk was especially high for male participants and following the death of the patient with cancer (adjusted HR, 1.48; 95% CI, 1.44-1.52) (eTable 7 in Supplement 1).

In the separate analysis of spouses with preexisting psychiatric morbidity, there was a higher risk of hospital visit for any psychiatric disorder for spouses of patients with cancer during the entire follow-up period (adjusted HR, 1.23; 95% CI, 1.20-1.25) and especially during the first year of follow-up (adjusted HR, 1.31; 95% CI, 1.27-1.35) compared with spouses of individuals without cancer (Figure 2B). In the subgroup analysis of spouses with a previous diagnosis of psychiatric disorders other than substance abuse, depression, anxiety, or stress-related disorders, being spouse of a patient with cancer was also associated with a higher risk of new-onset substance abuse, depression, anxiety, and stress-related disorders.

## Discussion

In this binational study in Denmark and Sweden, we reported a higher risk of any psychiatric disorder, as well as substance abuse, depression, anxiety, and stress-related disorders, among spouses of patients with cancer compared with spouses of individuals without cancer. The risk increase was especially high during the first year following cancer diagnosis and persisted during the entire follow-up. We further observed that the risk increase was more prominent among the spouses of patients diagnosed with a cancer type with poor prognosis or at advanced stages and when the patient died during the follow-up period. Finally, the risk increase was higher among spouses in the exposed group with preexisting psychiatric morbidity compared with exposed spouses without such vulnerability.

Our results are in line with the previous finding of increased psychiatric burden among spouses of patients with cancer.^12,13^ However, we expanded the evidence to a wider range of psychiatric disorders, with a detailed analysis of the temporal pattern over an extended period and addressed multiple characteristics of the spouses and patients with cancer. The greatest risk increase during the first year after cancer diagnosis was consistent with our previous finding that the risk of adverse health outcomes, eg, psychiatric disorders, cardiovascular disease, and suicide, was highest during the first year after diagnosis among the patients with cancer themselves.^34,35^

An increased risk of psychiatric disorders was observed among spouses of patients with cancer, regardless of the different characteristics of the spouses or the patients, implying a ubiquitous susceptibility to psychiatric disorders in this risk population. The higher risk increase observed in male spouses, especially after the death of the patient with cancer, might be attributed to both the lower baseline risk of psychiatric disorders among men and the fact that, given their different social roles and sources of emotional support, men might in general be less prepared for caregiving and coping with bereavement than women. Regardless, this finding suggests that male spouses of patients with cancer might be a high-risk group of mental illness. The greater risk increase noted in spouses in the exposed group aged 40 to 59 years and 60 to 79 years corroborates the finding by age at diagnosis of the patients with cancer in the present and previous studies.^34,35^ Consistent with earlier findings,^4^ spouses with a lower household income were more vulnerable to psychiatric disorders compared with spouses with a higher household income, highlighting the health inequity due to sociodemographic disparities. One possible explanation might be the greater amount of time spent on informal caregiving^3^ among spouses with lower household income.

The risk increase of psychiatric disorders among the spouses in the exposed group was higher for more aggressive cancer types and for cancers diagnosed at advanced stage. This is likely attributed to greater psychological distress experienced in receiving a diagnosis of a potentially fatal disease, higher burden of caregiving,^3^ and a greater possibility of bereavement. As previously suggested,^36^ we found that spouses experienced a higher risk increase of psychiatric disorders after the death of the patient. This finding also corroborates that the risk increase was greatest for cancers in the esophagus, lung, pancreas, and liver and biliary passages, which are cancers with poor prognosis and the highest documented rate of suicide.^37,38^ Finally, the association of being spouse of a patient with cancer with the risk of psychiatric disorders was found to be stronger among spouses with preexisting psychiatric morbidity, compared with spouses without such vulnerability, both immediately after cancer diagnosis and in the long run. These results, taken together, highlight the high-risk groups among the spouses of patients with cancer for potential targeted prevention, surveillance, and intervention.

Understanding and preventing psychiatric disorders among spouses of patients with cancer is important in terms of both preventing poor mental health among the spouses themselves and providing better support and care to individuals with cancer. Although studies have suggested that psychiatric disorders among caregivers could be predicted by the burden of care^4^ and the coping strategies and understanding of the disease among the patients being cared for,^7^ efforts on interventions to assist caregivers’ mental well-being are still in their infancy.^1,39^ Our study therefore reinforces the importance of awareness among families of patients with cancer, health care professionals, and society at large regarding the vulnerability of patients and their families, especially female patients with cancer, patients with more aggressive types or advanced stages of cancer, and during the first year after cancer diagnosis. The strengths of this study include the binational study population, large sample size, long and complete follow-up, and the prospectively and independently collected information on exposure and outcomes, which greatly reduce selection and recall biases, and increase internal and external validity.

### Limitations

This study has limitations. First, we were unable to identify spouses in the same way between Denmark and Sweden due to data availability. However, this differential definition is not likely to bias our results greatly due to similar associations between the 2 countries and between Danish individuals with or without a child. Second, surveillance bias might be a concern assuming that spouses of patients with cancer had greater access to health care than spouses in the unexposed group. However, it was suggested that spouses of patients with cancer had lower health care costs than the general population,^12^ possibly due to negligence of their own health. Third, we did not ascertain psychiatric disorders attended by primary care, as the registers used to define psychiatric disorders include only secondary health care. Indeed, approximately half of psychiatric disorders are managed in primary care settings in Sweden.^40^ Whether this would affect the exposed and unexposed groups differentially is uncertain, as spouses of patients with cancer might have greater or less access to specialist care as discussed previously. Regardless, it is important to emphasize that the results of our study are only generalizable to psychiatric disorders requiring secondary care. Fourth, information on education, smoking, BMI, CCI, and psychiatric history was not available for all participants. However, the missingness is due to administrative reasons and should not be differential in relation to the exposure or outcome. Regardless, the largely similar results observed between the analyses with or without adjustment for these factors argue against strong impact of such missingness. Fifth, we lacked information on whether the spouse lived with the patient with cancer, the cancer treatments, and availability of other caregivers. Such information would help to understand the reasons for the excess risk and shed light on potential interventions. Sixth, although we tried our best to control for potential confounding, we could not rule out residual confounding completely, due to the observational nature of the study.

## Conclusions

In this cohort study of 2 populations in Denmark and Sweden, spouses of patients with cancer showed an increased risk of psychiatric disorders requiring secondary care, both immediately after cancer diagnosis and during the decades thereafter. The risk increase was greater during the first year after cancer diagnosis, for fatal cancer, among male spouses, and among spouses with preexisting psychiatric morbidity. These results support the need of clinical awareness to prevent potential mental illness among the spouses of patients with cancer, especially in these high-risk groups.

## Notes

Supplement 1. **eMethods.** Proportional Hazard (PH) Assumption of Cox Models **eTable 1.** *International Classification of Diseases* (*ICD*) Codes for Cancer Subtypes in the Danish and Swedish Cancer Registers **eTable 2.** The Danish and Swedish Revisions of *International Classification of Diseases* (*ICD*) Codes for Psychiatric Disorders **eTable 3.** Baseline Characteristics of the Study Participants by Country **eTable 4.** Hazard Ratios (HRs) and 95% CIs of Any First-Onset Disorder in Relation to Being Spouse of a Patient With Cancer, Subgroup Analysis by Characteristics of the Patient **eTable 5.** Hazard Ratios (HRs) and 95% CIs of Any First-Onset Psychiatric Disorder in Relation to Being Spouse of a Patient With Cancer, With Further Adjustment for Highest Attained Education, Smoking Status, Body Mass Index, or Charlson Comorbidity Index **eTable 6.** Hazard Ratios (HRs) and 95% CIs of Any First-Onset Psychiatric Disorder in Relation to Being a Spouse of a Patient With Cancer, Stratified Analysis by Family Status **eTable 7.** Hazard Ratios (HRs) and 95% CIs of Any First-Onset Psychiatric Disorder in Relation to Being a Spouse of a Patient With Cancer, Stratified by Sex and Marital Status **eFigure.** Flow Chart of the Study Design

Supplement 2.
Data Sharing Statement
