# Supplementary material for: Risk of Psychiatric Disorders Among Spouses of Patients With Cancer in Denmark and Sweden
Source: JAMA Netw Open. 2023 Jan 5;6(1):e2249560. doi: 10.1001/jamanetworkopen.2022.49560 (PMC9857700; doi:10.1001/jamanetworkopen.2022.49560)
Supplement: Supplement 1. — eMethods. Proportional Hazard (PH) Assumption of Cox Models eTable 1. International Classification of Diseases (ICD) Codes for Cancer Subtypes in the Danish and Swedish Cancer Registers eTable 2. The Danish and Swedish Revisions of International Classification of Diseases (ICD) Codes for Psychiatric Disorders eTable 3. Baseline Characteristics of the Study Participants by Country eTable 4. Hazard Ratios (HRs) and 95% CIs of Any First-Onset Disorder in Relation to Being Spouse of a Patient With Cancer, Subgroup Analysis by Characteristics of the Patient eTable 5. Hazard Ratios (HRs) and 95% CIs of Any First-Onset Psychiatric Disorder in Relation to Being Spouse of a Patient With Cancer, With Further Adjustment for Highest Attained Education, Smoking Status, Body Mass Index, or Charlson Comorbidity Index eTable 6. Hazard Ratios (HRs) and 95% CIs of Any First-Onset Psychiatric Disorder in Relation to Being a Spouse of a Patient With Cancer, Stratified Analysis by Family Status eTable 7. Hazard Ratios (HRs) and 95% CIs of Any First-Onset Psychiatric Disorder in Relation to Being a Spouse of a Patient With Cancer, Stratified by Sex and Marital Status eFigure. Flow Chart of the Study Design [file jamanetwopen-e2249560-s001.pdf]

Supplemental Online Content

Hu K, Liu Q, László KD, et al. Risk of psychiatric disorders among spouses of patients with cancer in Denmark and Sweden. *JAMA Netw Open.* 2023;6(1):e2249560.
doi:10.1001/jamanetworkopen.2022.49560

eMethods. Proportional Hazard (PH) Assumption of Cox Models

eTable 1. *International Classification of Diseases* (*ICD*) Codes for Cancer Subtypes in the Danish and Swedish Cancer Registers

eTable 2. The Danish and Swedish Revisions of *International Classification of Diseases* (*ICD*) Codes for Psychiatric Disorders

eTable 3. Baseline Characteristics of the Study Participants by Country

eTable 4. Hazard Ratios (HRs) and 95% CIs of Any First-Onset Disorder in Relation to Being Spouse of a Patient With Cancer, Subgroup Analysis by Characteristics of the Patient

eTable 5. Hazard Ratios (HRs) and 95% CIs of Any First-Onset Psychiatric Disorder in Relation to Being Spouse of a Patient With Cancer, With Further Adjustment for Highest Attained Education, Smoking Status, Body Mass Index, or Charlson Comorbidity Index

eTable 6. Hazard Ratios (HRs) and 95% CIs of Any First-Onset Psychiatric Disorder in Relation to Being a Spouse of a Patient With Cancer, Stratified Analysis by Family Status

eTable 7. Hazard Ratios (HRs) and 95% CIs of Any First-Onset Psychiatric Disorder in Relation to Being a Spouse of a Patient With Cancer, Stratified by Sex and Marital Status

eFigure. Flow Chart of the Study Design

This supplemental material has been provided by the authors to give readers additional information about their work.

© 2023 Hu K et al. *JAMA Network Open.*

eMethods. Proportional Hazard (PH) Assumption of Cox Models

For all Cox proportional hazards models, we plotted the Schoenfeld residuals against time for the exposure variables and found the residuals to largely scatter around zero. No major deviation from the proportional hazard assumption was therefore detected for any of the exposure variables. As exposure variables are our primary interest and violations of the assumption for these variables are arguably more severe and likely to cause more bias, than violations of the assumption for covariates, it is a reasonable pragmatic compromise to only test the proportionality assumption for the exposure variables.

© 2023 Hu K et al. *JAMA Network Open.*

eTable 1. ***International Classification of Diseases* (*ICD*) Codes for Cancer Subtypes in the Danish and Swedish Cancer Registers**

Cancer site/type	ICD-7	ICD-10
**Any cancer**	140-207	C00-C97
**Bladder**	181	C67
**Breast**	170	C50
**Central nervous system**	193	C70-C72
**Cervix uteri**	171	C53
**Colon**	153	C18
**Corpus uteri**	172	C54
**Esophagus**	150	C15
**Hematological malignancy**	200-207	C81-C96
**Kidney**	180	C64-C66
**Liver and biliary passages**	155	C22-C24
**Lung (incl. trachea and bronchus)**	162-163	C33-C34
**Melanoma**	190	C43
**Non-melanoma skin**	191	C44
**Ovary**	175	C56
**Pancreas**	157	C25
**Prostate**	177	C61
**Rectum**	154	C20
**Stomach**	151	C16
**Thyroid and other endocrine gland**	194-195	C73-C75
**Upper aerodigestive tract**	140-141, 143-148, 161	C00-C07, C09-C14, C32

To identify the cancer patients, ICD-7 (1943–1977) and ICD-10 (1978-) were used in Danish Cancer Register and ICD-7 (1958-) was used in Swedish Cancer Register.

eTable 2. The Danish and Swedish Revisions of *International Classification of Diseases* (*ICD*) Codes for Psychiatric Disorders

Diseases	Denmark	Sweden
**Any psychiatric disorder**	ICD-8: 291, 295-315<br>ICD-10: F10-F99	ICD-8: 291, 295-315<br>ICD-9: 291, 292, 295-319<br>ICD-10: F10-F99
**Substance abuse**	ICD-8: 291, 303, 304<br>ICD-10: F10-F19	ICD-8: 291, 303, 304<br>ICD-9: 291, 292, 303-305<br>ICD-10: F10-F19
**Depression**	ICD-8: 29609, 29629, 29699, 29809, 30049<br>ICD-10: F32, F33	ICD-8: 296.0, 296.2, 298.0, 300.4<br>ICD-9: 296B, 296D, 298A, 300E, 311<br>ICD-10: F32, F33
**Anxiety**	ICD-8: 3000, 3002<br>ICD-10: F40, F41	ICD-8: 300.0, 300.2<br>ICD-9: 300A, 300C<br>ICD-10: F40, F41
**Stress-related disorders**	ICD-8: 307<br>ICD-10: F43	ICD-8: 307<br>ICD-9: 308-309<br>ICD-10: F43

ICD codes were used to identify psychiatric disorders in the Danish Patient Register, Civil Registration System, and Psychiatric Central Register, as well as in the Swedish Patient and Causes of Death Register. ICD-8 was implemented in Denmark during 1977-1993 and replaced by ICD-10 since 1994. For Swedish revisions of ICD codes, ICD-8 was used during 1968-1986, replaced by ICD-9 during 1987-1996 and replaced by ICD-10 since 1997.

eTable 3. Baseline Characteristics of the Study Participants by Country

Characteristics	Denmark		Sweden	
	**Spouses of cancer patients (exposed)**	**Spouses of cancer-free individuals (unexposed) a**	**Spouses of cancer patients (exposed)**	**Spouses of cancer-free individuals (unexposed) a**
**Total number**	384,553	1,922,757	161,768	808,817
	**Participants, No. (%)**	**Participants, No. (%)**	**Participants, No. (%)**	**Participants, No. (%)**
**Sex**				
Male	172,662 (44.9)	863,302 (44.9)	78,800 (48.7)	393,985 (48.7)
Female	211,891 (55.1)	1,059,455 (55.1)	82,968 (51.3)	414,832 (51.3)
**Age at cohort entry, Median [IQR], years**	63 [54-70]	63 [54-70]	54 [45-61]	54 [45-61]
**Age at cohort entry, years**				
18-39	16,662 (4.3)	83,445 (4.3)	23,874 (14.8)	119,370 (14.8)
40-59	131,491 (34.2)	657,813 (34.2)	87,824 (54.3)	439,120 (54.3)
60-79	215,603 (56.1)	1,077,481 (56.0)	49,749 (30.8)	248,734 (30.1)
80-104	20,797 (5.4)	104,018 (5.4)	321 (0.2)	1,593 (0.2)
**Calendar year of cohort entry**				
1973-1989	29,945 (7.8)	149,725 (7.8)	13,103 (8.1)	65,512 (8.1)
1990-1999	98,732 (25.7)	493,660 (25.7)	27,651 (17.1)	138,254 (17.1)
2000-2009	147,770 (38.4)	738,842 (38.4)	65,657 (40.6)	328,271 (40.6)
2010-2016	108,106 (28.1)	540,530 (28.1)	55,357 (34.2)	276,780 (34.2)
**Country of residence**				
Denmark	192,992 (50.2)	1,052,899 (54.8)	26,668 (16.5)	124,752 (15.4)
Sweden	52,211 (13.6)	242,485 (12.6)	57,993 (35.9)	285,718 (35.3)
**Household income** [b]	139,020 (36.2)	623,021 (32.4)	75,759 (46.8)	391,420 (48.4)
Below the lowest tertile	330 (0.1)	4,352 (0.2)	1,348 (0.8)	6,927 (0.9)
Between the lowest and middle tertile				
Above the highest tertile	39,497 (10.3)	168,329 (8.8)	8,167 (5.0)	40,004 (4.9)
Unknown	345,056 (89.7)	1,754,428 (91.3)	153,601 (95.0)	768,813 (95.1)
**History of cancer**				
Yes	140,551 (36.6)	717,166 (37.3)	31,929 (19.7)	160,066 (19.8)
No	156,315 (40.7)	763,571 (39.7)	87,997 (54.4)	440,275 (54.4)
**Years of education**	63,295 (16.5)	308,634 (16.1)	26,481 (16.4)	131,622 (16.3)
0-9	24,392 (6.3)	133,386 (6.9)	1,561 (9.5)	76,854 (9.5)

IQR, interquartile range.
a Individually matched by sex, year of birth, and country of residence. Date of cohort entry was the date of cancer diagnosis for the exposed spouses.
b Household income were classified into three groups according to the tertile distribution of each 10-year intervals in each country.

© 2023 Hu K et al. *JAMA Network Open.*

eTable 4. Hazard Ratios (HRs) and 95% CIs of Any First-Onset Disorder in Relation to Being Spouse of a Patient With Cancer, Subgroup Analysis by Characteristics of the Patient

**Characteristics of the cancer patient**	**HR (95% CI)** [a]
**Age at diagnosis, years**	
18-39	1.04 (1.00-1.07)
40-59	1.18 (1.16-1.20)
60-79	1.14 (1.13-1.17)
80-104	1.06 (1.11-1.11)
**Cancer stage** [b]	
Localized	0.98 (0.95-1.01)
Regional spread or advanced	1.31 (1.26-1.36)
Unknown	1.16 (1.14-1.17)

[a] Estimated from Cox models, adjusting for sex, age at cohort entry, calendar year at cohort entry, country of residence, household income, and history of cancer.

[b] Analysis restricted to Danish participants with a spouse diagnosed with cancer from 2004 onward and their individually matched unexposed spouses. TNM information was used to classify cancer stage into localized (any T/N0/M0), regional spread or advanced (any T/N+/M0 or any T/any N/M+), and unknown stage according to the European Network of Cancer Registries Condensed TNM Scheme. Cancers with missing or unknown TNM stage and cancer of the central nervous system were classified as "Unknown". Haematological malignancies were excluded from the analysis.

© 2023 Hu K et al. *JAMA Network Open.*

eTable 5. Hazard Ratios (HRs) and 95% CIs of Any First-Onset Psychiatric Disorder in Relation to Being Spouse of a Patient With Cancer, With Further Adjustment for Highest Attained Education, Smoking Status, Body Mass Index, or Charlson Comorbidity Index

Additional adjustment	HR (95% CI)
Highest attained education [a]	1.15 (1.14-1.17)
Smoking status [b]	1.11 (1.07-1.16)
Body mass index [c]	1.15 (1.10-1.21)
Charlson Comorbidity Index [d]	1.16 (1.14-1.17)

[a] HRs and 95% CIs were estimated from Cox models, adjusting for sex, age at cohort entry, calendar year at cohort entry, country of residence, household income, history of cancer, and highest attained education (alternatively smoking status, body mass index or Charlson Comorbidity Index).

[b] Smoking status during the first pregnancy was retrieved from the Danish and Swedish Medical Birth Register (only available for pregnant women) and available since 1991 in Denmark and 1982 in Sweden.

[c] Body mass index (BMI) during the first pregnancy was retrieved from the Danish and Swedish Medical Birth Register (only available for pregnant women) and available since 2004 in Denmark and during 1982-1989 and since 1992 in Sweden.

[d] limited to Danish participants.

© 2023 Hu K et al. *JAMA Network Open.*

eTable 6. Hazard Ratios (HRs) and 95% CIs of Any First-Onset Psychiatric Disorder in Relation to Being a Spouse of a Patient With Cancer, Stratified Analysis by Family Status

**Family status**	**HR (95% CI)** [a]
**Marital status** [b]	
No change	1.05 (1.03-1.07)
Divorced	1.03 (1.01-1.05)
Widowed	1.29 (1.27-1.31)
**Sharing a biological child** [c]	
Yes	1.20 (1.19-1.22)
No	1.15 (1.11-1.20)

[a] Estimated from Cox models, adjusting for sex, age at cohort entry, calendar year at cohort entry, country of residence, household income, and history of cancer.
[b] Marital status was treated as time-varying variable, considering information at the time of cohort entry as well as during follow-up. Follow-up was split at the change of marital status (e.g., divorce or death of the cancer patient).
[c] Limited to Danish participants and treated as a fixed variable, considering information at the time of cohort entry. There were in total 1,861,180 spouses with at least one child and 446,130 spouses without. The incidence rate per 1000 person-years was 6.0 among spouses who had had a child and 8.4 among spouses who had not.

© 2023 Hu K et al. *JAMA Network Open.*

eTable 7. Hazard Ratios (HRs) and 95% CIs of Any First-Onset Psychiatric Disorder in Relation to Being a Spouse of a Patient With Cancer, Stratified by Sex and Marital Status

	**Males**	**Females**
	**HR (95% CI)** [a]	**HR (95% CI)** [a]
**Marital status** [b]		
No changes	1.06 (1.04-1.08)	1.04 (1.02-1.06)
Divorced	1.04 (1.02-1.07)	1.03 (1.00-1.05)
Widowed	1.48 (1.44-1.52)	1.18 (1.16-1.21)

[a] Estimated from Cox models, adjusting for sex, age at cohort entry, calendar year at cohort entry, country of residence, household income, and history of cancer.

[b] Marital status was treated as time-varying variable, considering information at the time of cohort entry as well as during follow-up. Follow-up was split at the change of marital status (e.g., divorce or death of the cancer patient).

© 2023 Hu K et al. *JAMA Network Open.*

eFigure. Flow Chart of the Study Design

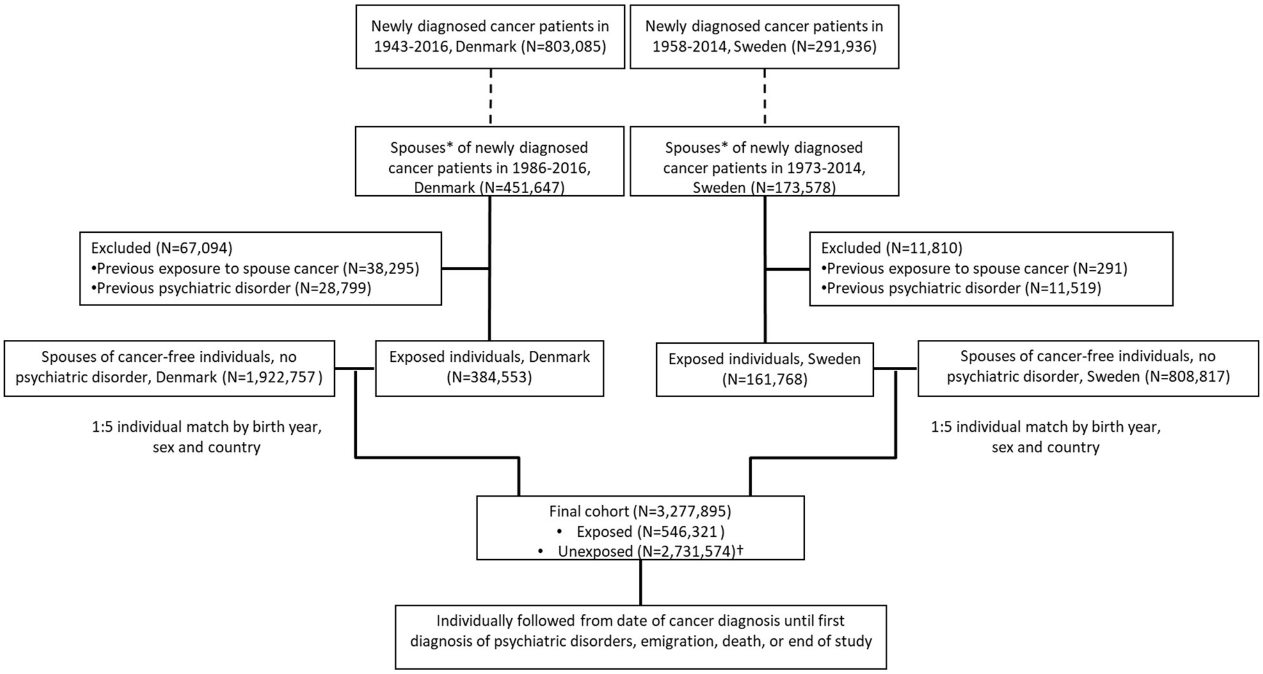

This study was designed within the Danish-Swedish joint project of stressful life events, which includes general Danish population born during 1878-2016, and Swedish population born during 1973-2014 together with their parents.

*The spouses were defined using spousal link for the Danish population, and shared biological child and marital status for Swedish population.

† The number of unexposed spouses is not equal to 5 times the number of exposed spouses due to a limited number of eligible spouses born in earlier years (<1932).

© 2023 Hu K et al. *JAMA Network Open.*
