# Supplementary material for: Risk of Psychiatric Disorders Among Spouses of Patients With Cancer in Denmark and Sweden
Source: JAMA Netw Open. 2023 Jan 5;6(1):e2249560. doi: 10.1001/jamanetworkopen.2022.49560 (PMC9857700; doi:10.1001/jamanetworkopen.2022.49560)
Supplement: Supplement 2. — Data Sharing Statement [file jamanetwopen-e2249560-s002.pdf]

Data Sharing Statement

Hu. Risk of Psychiatric Disorders Among Spouses of Patients With Cancer in Denmark and Sweden. *JAMA Netw Open*. Published January 05, 2023.
doi:10.1001/jamanetworkopen.2022.49560

Data
Data available: No

Additional Information
Explanation for why data not available: We are not able to make the data available due to Danish and Swedish laws. However, it is possible to apply the same data for research from Danish and Swedish registers.
